# Estimated hospitalizations attributed to norovirus and rotavirus infection in Canada, 2006–2010

**DOI:** 10.1017/S0950268815000734

**Published:** 2015-05-20

**Authors:** V. K. MORTON, M. K. THOMAS, S. A. McEWEN

**Affiliations:** 1 Centre for Foodborne, Environmental and Zoonotic Infectious Diseases, Public Health Agency of Canada, Guelph, Ontario Canada; 2 University of Guelph, Department of Population Medicine, Guelph, Ontario Canada

**Keywords:** Norwalk agent and related viruses, rotavirus

## Abstract

Enteric viruses including norovirus and rotavirus are leading causes of gastroenteritis in Canada. However, only a small number of clinical cases are actually tested for these pathogens leading to systematic underestimation of attributed hospitalizations in administrative databases. The objective of this analysis was to estimate the number of hospitalizations due to norovirus and rotavirus in Canada. Hospitalization records for acute gastroenteritis-associated discharges at all acute-care hospitals in Canada between 2006 and 2011 were analysed. Cause-unspecified gastroenteritis hospitalizations were modelled using age-specific negative binomial models with cause-specified gastroenteritis admissions as predictors. The coefficients from the models were used to estimate the number of norovirus and rotavirus admissions. The total annual hospitalizations for rotavirus were estimated to be between 4500 and 10 000. Total annual hospitalizations for norovirus were estimated to be between 4000 and 11 000. The mean total annual cost associated with these hospitalizations was estimated to be at least $16 million for rotavirus and $21 million for norovirus (all figures in Canadian dollars). This study is the first comprehensive analysis of norovirus and rotavirus hospitalizations in Canada. These estimates provide a more complete assessment of the burden and economic costs of these pathogens to the Canadian healthcare system.

## INTRODUCTION

Enteric illness is an important cause of morbidity and mortality worldwide [1]. In Canada, norovirus and rotavirus are two of the leading causes of acute gastroenteritis (AGE). Recent estimates indicate that about 3·4 million norovirus and 850 000 rotavirus cases occur annually [2]. Although these illnesses are common, cases are rarely confirmed through laboratory testing because diagnosis does not change case management [3, 4]. As a result of this limited testing, the incidence of these viruses tends to be systematically underrepresented in surveillance databases, including hospital records. In the absence of reliable information concerning hospitalizations it is difficult to assess their burden on the healthcare system, and to evaluate the potential costs and benefits of alternative public health policies.

Both norovirus and rotavirus are enteric viruses that can cause AGE; however, infections caused by these agents exhibit different epidemiological characteristics. Norovirus typically causes mild, self-limiting gastroenteritis in individuals of all ages and is most prevalent in the winter months. The virus is infectious at very low doses and is extremely stable in the environment, spreading easily from person to person [5]. Outbreaks occur frequently in closed settings such as schools, hospitals, long-term care facilities and cruise ships [6–9]. Although the virus can infect individuals of all ages, the impacts of infection tend to be greater in the elderly and individuals with underlying health conditions who are more likely to develop complications, and require hospitalization [10, 11].

Rotavirus infections occur in individuals of all ages, but illness in adults tends to be mild or asymptomatic [12]. Severe illness is most common in young children aged <5 years and can result in dehydration requiring medical attention or hospitalization [13]. Rotavirus infections are extremely common worldwide; with about 95% of children contracting the illness by age 5 years, and as a result the burden of this illness on the healthcare system is substantial, in both developed and developing countries [14]. To lessen this burden, two vaccines have recently been developed for rotavirus. These vaccines have been shown to significantly decrease hospitalization rates for young children [15, 16].

A recent study used indirect attribution from regression modelling techniques to estimate the incidence of norovirus and rotavirus hospitalization in the United States [17]. Similar to Canada, testing for enteric viruses is limited in the United States and many resulting hospitalizations are not laboratory-confirmed, therefore these cases are classified as unspecified AGE since the causative agent is unknown. By examining hospitalization records attributed to AGE, Lopman *et al*. [17], were able to attribute portions of unspecified AGE hospitalizations to these viruses and were able to estimate the total number of hospitalizations and costs attributed to each virus.

The objective of the present study was to apply similar methods to examine Canadian hospital administration records and develop Canadian-specific estimates of hospitalization rates, and the associated costs for norovirus and rotavirus infection in different age groups [17].

## METHODS

### Data sources

Hospitalization data were obtained from the Hospital Morbidity Database maintained by the Canadian Institute for Health Information (CIHI-HMDB) [18]. This database captures administrative, clinical and demographic information on all patient discharges from all acute-care hospitals across Canada. Clinical diagnoses are recorded using the International Statistical Classification of Diseases and Related Health Problems Tenth Revision, Canada (ICD-10-CA) coding standards. Up to 35 diagnoses are recorded in each patient discharge record. The first diagnosis, also known as the most responsible diagnosis, records the diagnosis most responsible for the patient's stay in the hospital. Remaining diagnoses are given a diagnosis type, including pre-admission comorbidity, post-admission comorbidity or secondary diagnosis.

CIHI-HMDB records with an admission date between 1 April 2006 and 31 March 2011 and at least one AGE diagnosis code (Table 1) within the first 16 diagnosis code positions were extracted. Entries were categorized using a hierarchy of specificity with specified pathogens' codes given priority over cause-unspecified codes. Records with a specific pathogen code (e.g. *C. difficile*) and a cause-unspecified gastroenteritis code were categorized based on the pathogen code. Entries with more than one AGE category of pathogen code (e.g. *C. difficile* and viral AGE code) were excluded from analysis, as they could not be categorized.

**Table 1.** Descriptive statistics and associated diagnostic codes for acute gastroenteritis discharges, 2006–2011 CIHI-HMDB*

Cause	ICD-10 codes	No. of patients	Median age (yr)	Median length of stay (days)	% Males	% Deaths†	% Post-admission‡
Bacteria	A00–A04.6, A04.8–A05.9	13 712	51	4	49	2·9	4·9
*Clostridium difficile*	A04.7	83 606	76	17	44	17·5	39·7
Parasite	A06–A06.2, A06.9–A07.9	1017	42	5	56	3·1	3·3
Rotavirus	A08.0	8976	1	2	54	0·2	6·1
Norovirus	A08.1	3264	77	11	42	7·3	46·2
Adenovirus	A08.2	635	1	3	60	2·1	10·1
Other virus	A08.3	2906	4	2	49	1·7	9·5
Unspecified virus	A08.4	46 099	31	2	43	1·0	5·6
Unspecified gastroenteritis	A09–A09.9, K52.8–K52.9	240 137	61	4	41	3·7	12·9

* Canadian Institutes of Health Information – Hospital Morbidity Database.

† Percent of total discharges where the discharge disposition was listed as deceased.

‡ Percent of total discharges where diagnostic code of interest was classified as developing post-admission to hospital.

Descriptive statistics were used to describe discharges by pathogen category. Death rates were calculated based on the discharge disposition recorded (i.e. status of patient at time of discharge). Incidence rates were calculated based on, age-stratified population estimates for Canada (2006–2010) obtained from Statistics Canada [19].

Reports of laboratory-confirmed clinical cases of enteric viruses from 2006 to 2011 were obtained from the National Enteric Surveillance Program (NESP) [20]. The NESP collects weekly counts of enteric pathogens from all 10 central provincial public health laboratories across Canada. This provided an independent dataset with which to compare the estimates developed.

### Statistical analysis

The number of norovirus- and rotavirus-associated hospitalizations were estimated using an approach presented by Lopman *et al.* [17]. This approach assumes that a proportion of cause-unspecified gastroenteritis (unspecified gastroenteritis and unspecified virus) hospitalizations are due to norovirus or rotavirus infections. In order to estimate the number of hospitalizations, models were built to account for hospitalizations that likely occurred due to bacterial pathogens (including *C. difficile*), viral and parasitic pathogens among the cause-unspecified hospitalizations. Specifically, the numbers of monthly cause-unspecified diagnoses were modelled as a function of the monthly admissions associated with bacterial, viral, parasitic and *C. difficile* infections using the following negative binomial generalized linear model:

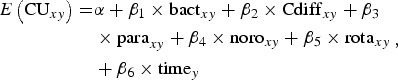

where *x* represents the age group and *y* the month. Hospitalization records were modelled based on the month of admission as this was thought to be more reflective of the seasonal pattern of infection compared to month of discharge. A time parameter was also included in the model to account for any underlying hospitalization trends over time. Models were fit using an identity link to ensure that regression coefficients were on a linear scale. A separate model was generated for each of five age groups (0–4, 5–17, 18–64, 65–84, ⩾85 years). Coefficient values from the models for norovirus and rotavirus were multiplied by the monthly hospitalization count for norovirus and rotavirus, respectively, to estimate the number of cause-unspecified cases attributed to the two viruses. Confidence intervals around the coefficients were similarly used to develop 95% confidence intervals around the estimates. To determine the overall number of hospitalizations, estimates from the model were combined with the coded norovirus and rotavirus entries from CIHI-HMDB.

Analysis was conducted using SAS version 9.3 (SAS Institute Inc., USA) and Excel 2010 (Microsoft Corporation, USA).

### Hospitalization costs

Mean annual hospitalization costs by age group for discharges with most responsible diagnostic code of norovirus (A08.1) or rotavirus (A08.0) were obtained for 2006 to 2008 [21]. These costs are calculated by attributing a proportion of the total hospital expenditure to each hospitalization stay. As a result, these values include all expenses associated with a hospital stay such as administrative costs, drugs and medical supplies, staff salaries and hospital maintenance. These age-specific costs were modelled using a triangular distribution where the minimum, most likely and maximum values were the lowest, mean and highest annual cost per case during this time-frame; and multiplied by a cumulative distribution of age-specific total annual admissions for each of norovirus and rotavirus. This method helps to account for uncertainty and variability of the inputs. Estimates were generated using Monte Carlo simulations (10 000 iterations) with @Risk software (Palisade Corporation, USA). All dollar figures are reported in Canadian currency and adjusted to 2008 values to account for inflation using the Bank of Canada Inflation Calculator (http://www.bankofcanada.ca/rates/related/inflation-calculator/).

## RESULTS

During the study period 401 364 discharges were identified with at least one diagnostic code of interest within the first 16 diagnoses; however, 1051 (0·2%) of these discharges had more than one category of diagnostic code of interest and were removed from the analysis. Discharges were categorized based on the type of causative agent (Table 1). Median age of infected patients ranged from 1 year (rotavirus and adenovirus) to 76–77 years (*C. difficile* and norovirus). Measures of severity, such as length of stay and death rate, were highest for *C. difficile* and norovirus. The percent of post-admission diagnoses was also highest for norovirus (46%), a measure of nosocomial transmission. The percent of norovirus infections diagnosed post-admission ranged considerably by age, from 17% in those aged 0–17 years to 52–54% in those aged ⩾65 years. Similarly for rotavirus infection, about 5% of cases were nosocomial in individuals aged 0–17 years, while 27–34% of cases in individuals aged ⩾65 years were classified as post-admission conditions (data not shown).

**Fig. 1.**
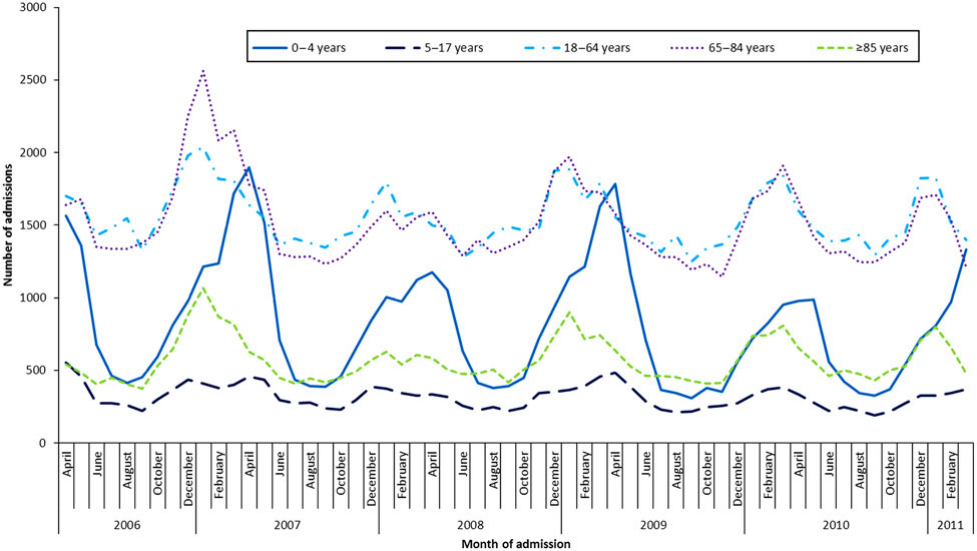
Seasonal pattern of cause-unspecified gastroenteritis hospitalizations, month and year of admission, by age group, 2006–2011, Canadian Institutes of Health Information – Hospital Morbidity Database.

In total, there was a mean annual rate of 24 hospital discharges due to AGE illnesses per 10 000 in Canada (Table 2). The percentages of discharges specifically attributed to norovirus and rotavirus infection were low at 1% and 2%, respectively. However, the majority (60%) of AGE hospitalizations were due to unspecified agents, with an additional 11% attributed to unspecified viruses. The monthly distribution of cause-unspecified gastroenteritis shows a seasonal pattern (Fig. 1). In the older age groups (>18 years) the number of admissions peaks around January each year, which is consistent with the known seasonal patterns of norovirus in Canada [22]. In the youngest age group (0–4 years), a small increase occurs in January with a considerably larger peak around April, consistent with the seasonality of rotavirus infection in Canada [23].

**Table 2.** Mean annual total and incidence rate of gastroenteritis hospitalizations by pathogen category for 2006–2011, CIHI-HMDB*

	Most responsible diagnosis			Other diagnosis (2–16 diagnostic codes)		
Cause	Mean annual total	%	Mean annual incidence rate (per 10 000)	Mean annual total	%	Mean annual incidence rate (per 10 000)
Bacteria	1884	5	0·56	2742	3	0·81
*Clostridium difficile*	5568	14	1·65	16 940	21	5·04
Parasite	80	0	0·02	202	0	0·06
Rotavirus	1456	4	0·43	1780	2	0·53
Norovirus	184	0	0·05	587	1	0·17
Adenovirus	87	0	0·03	130	0	0·04
Other virus	369	1	0·11	562	1	0·17
Unspecified virus	6274	16	1·87	8986	11	2·67
Unspecified gastroenteritis	23 135	59	6·88	47 826	60	14·22
Total	39 035		11·61	79 754		23·71

* Canadian Institutes of Health Information – Hospital Morbidity Database.

**Fig. 2.**
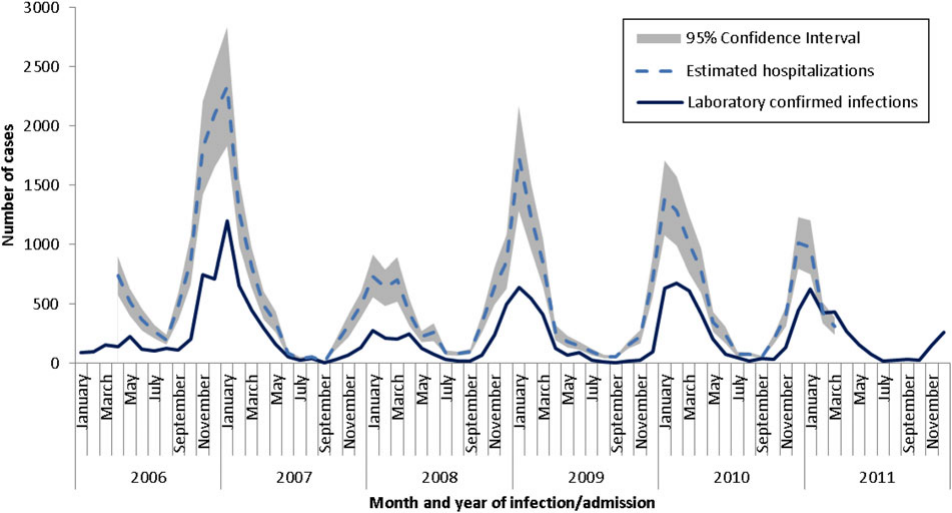
Seasonal distribution of laboratory-confirmed norovirus infections reported to the National Enteric Surveillance Program (NESP) and estimated norovirus hospitalizations by month and year of admission, 2006–2011.

**Fig. 3.**
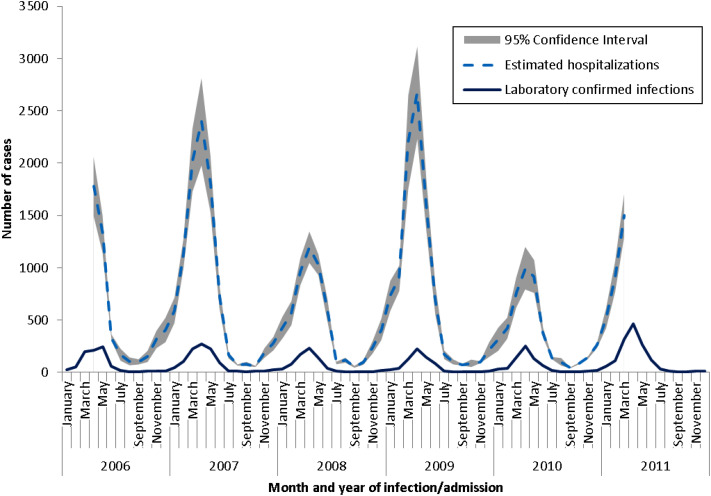
Seasonal distribution of laboratory-confirmed rotavirus infections reported to the National Enteric Surveillance Program (NESP) and estimated rotavirus hospitalizations by month and year of admission, 2006–2011.

Overall, the model estimated that norovirus and rotavirus infections account for about 11% and 10% of the total AGE cause-unspecified hospitalizations, respectively. Model predictions combined with coded hospitalizations resulted in total annual hospitalizations of between 4097 and 11 077 for norovirus infection and 4540–9931 for rotavirus infection (Tables 3 and 4). The monthly distribution pattern of the estimates corresponded closely with the seasonal trend reported in NESP for norovirus and rotavirus, respectively (Figs 2 and 3). The number of isolates reported through this programme was substantially underestimated compared to the estimated burden of illness. However, the number of laboratories reporting through NESP is consistent, therefore the seasonal trend in reported cases is reflective of the true seasonal trends [24]. Comparison of the monthly estimates with independent data from NESP provided additional evidence that the hospitalization estimates obtained from modelling were reflective of observed trends in Canada.

**Table 3.** Estimated (including known coded hospitalizations) norovirus-associated hospitalizations and incidence per 100 000 people, by age group and year, 2006–2010

	Number of hospitalizations (95% CI)						Incidence per 100 000 persons (95% CI)					
Year	0–4 yr	5–17 yr	18–64 yr	65–84 yr	⩾85 yr	All ages	0–4 yr	5–17 yr	18–64 yr	65–84 yr	⩾85 yr	All ages
2006/2007	1321	157	3116	4102	2380	11 077	95	1	59	107	446	34
	(878–1765)	(24–290)	(2421–3812)	(3353–4852)	(1967–2793)	(8643– 13 512)	(63–127)	(0–1)	(46–73)	(88–127)	(368–523)	(26–41)
2007/2008	1021	140	966	1097	873	4097	72	1	19	28	155	12
	(678–1364)	(22–259)	(750–1181)	(897–1298)	(721–1024)	(3068–5126)	(48–97)	(0–1)	(14–23)	(23–33)	(128–181)	(9–16)
2008/2009	1321	190	1975	1982	1067	6535	92	1	39	50	179	20
	(878–1765)	(29–351)	(1534–2416)	(1620–2343)	(882–1252)	(4943–8127)	(61–123)	(0–2)	(30–47)	(41–59)	(148–210)	(15–24)
2009/2010	1121	165	1639	2203	1239	6366	76	1	32	54	198	19
	(745–1498)	(25–305)	(1273–2004)	(1800–2605)	(1024–1454)	(4867–7865)	(51–102)	(0–1)	(25–39)	(44–64)	(163–232)	(14–23)
Mean	1196	163	1924	2346	1390	7019	84	1	37	60	244	21
	(795–1598)	(25–301)	(1494–2353)	(1917–2774)	(1148–1631)	(5380–8658)	(56–112)	(0–1)	(29–45)	(49–71)	(202–287)	(16–26)

CI, Confidence interval.

**Table 4.** Estimated (including known coded hospitalizations) rotavirus-associated hospitalizations and incidence per 100 000 people, by age group and year, 2006–2010

	Number of hospitalizations (95% CI)						Incidence per 100 000 persons (95% CI)					
Year	0–4 yr	5–17 yr	18–64 yr	65–84 yr	⩾85 yr	All ages	0–4 yr	5–17 yr	18–64 yr	65–84 yr	⩾85 yr	All ages
2006/2007	7525	930	640	674	162	9931	542	4	12	18	30	30
	(7002–8047)	(695–1165)	(148–1132)	(317–1032)	(34–289)	(8196– 11 666)	(504–579)	(3–5)	(3–22)	(8–27)	(6–54)	(25–36)
2007/2008	4395	493	180	491	65	5622	312	2	3	13	11	17
	(4089–4700)	(368–617)	(42–318)	(230–751)	(14–116)	(4743–6502)	(290–333)	(2–3)	(1–6)	(6–19)	(2–20)	(14–20)
2008/2009	7217	960	400	981	259	9816	501	4	8	25	43	29
	(6716–7718)	(717–1203)	(93–707)	(461–1501)	(54–463)	(8041– 11 592)	(466–536)	(3–5)	(2–14)	(12–38)	(9–78)	(24–35)
2009/2010	3201	344	360	521	113	4540	217	2	7	13	18	13
	(2979–3423)	(257–431)	(84–637)	(245–798)	(24–203)	(3588–5491)	(202–232)	(1–2)	(2–13)	(6–19)	(4–32)	(11–16)
Mean	5584	682	395	667	150	7477	393	3	8	17	26	23
	(5197–5972)	(509–854)	(92–698)	(313–1020)	(31–268)	(6142–8813)	(366–420)	(2–4)	(2–13)	(8–26)	(5–46)	(19–27)

CI, Confidence interval.

Mean annual costs associated with norovirus hospitalizations in Canada were estimated at $20.8 million [90% credible interval (CrI) $14.9–$28.9 million] (Table 5). A majority (69%) of the costs were associated with hospitalizations in individuals aged ⩾65 years. The hospitalization costs were slightly lower for rotavirus infections, with a mean annual cost of $16.1 million (90% CrI $11.0–$22.3 million). Most of these costs (73%) were due to hospitalizations in the <5 years age group.

**Table 5.** Estimated annual hospital costs associated with norovirus and rotavirus infection, by age group, and seasonal years 2006/2007–2009/2010

	Norovirus		Rotavirus	
Age group (yr)	Cost per hospitalization, range	Annual cost, mean (90% CrI)	Cost per hospitalization, range	Annual cost, mean (90% CrI)
0–4	2354–2959	3 002 786	2215–2409	11 695 509
		(2 515 153–3 611 199)		(7 301 687–17 415 599)
5–17	1021–1879	212 572	2118–2327	1 358 122
		(161 926–279 306)		(757 824–2 166 385)
18–64	1763–2070	3 223 461	1536–2621	683 532
		(1 818 970–15 535 059)		(325 802–1 214 375)
65–84	3871–4940	8 273 017	2635–3681	1 808 555
		(4 323 058–1 535 059)		(1 336 943–2 762 153)
⩾85	4771–5485	6 126 561	1586–7150	571 448
		(4 333 677–10 963 642)		(215 329–1 191 148)
Total		20 838 397		16 117 165
		(14 934 994–28 919 793)		(10 987 357–22 325 928)

CrI, Credible interval.

All values are in Canadian dollars (adjusted to 2008 value).

## DISCUSSION

We estimate that on average 7000 and 7500 hospitalizations annually in Canada involved norovirus and rotavirus infection, respectively. These estimates represent a substantial increase compared to the number of hospitalizations with norovirus- or rotavirus-specific codes in hospital records; four times greater for rotavirus-related and 11 times greater for norovirus-related reported hospitalizations accounted for in hospital records. Based on these figures, we estimate that norovirus and rotavirus infections combined cost the Canadian healthcare system up to $37 million annually in direct hospital costs. Hospital records also indicate that a substantial proportion of norovirus cases are nosocomial.

In order to develop these estimates, modelling techniques were employed, based on the assumption that proportions of unspecified gastroenteritis hospitalizations are due to infection with norovirus and rotavirus. The results obtained are consistent with the known epidemiology of these enteric infections. For instance the estimated monthly seasonality corresponds well with the known seasonal distribution of infection with each virus: norovirus peaking in early winter and rotavirus a few months later in early spring [22, 23]. Moreover, the age distribution of the estimated hospitalizations is consistent with known infections due to these agents; the majority of rotavirus-associated hospitalizations were in the <5 years age group, while norovirus hospitalizations are more common in the elderly [10, 25].

The study by Lopman *et al.* [17] which served as a model for this work, calculated comparable annual hospitalization rates for both viruses; 24/100 000 for norovirus and 29/100 000 for rotavirus compared to our estimates of 21/100 000 and 23/100 000, respectively. One other study from The Netherlands also estimated the overall burden of norovirus, and reported an estimated annual hospitalization rate of 12/100 000 in the general population [26]. This estimate was, however, limited to community-acquired cases. Our estimate is higher but if we consider that about 46% of norovirus are nosocomial, our estimate for community-acquired cases would be 11/100 000 which is consistent with the estimate from The Netherlands.

Although few studies estimate the burden of norovirus hospitalization, numerous studies have been conducted to estimate the hospitalization rate for rotavirus infection in the 0–5 years age group. For example, two Canadian studies reported annual hospitalization rates of 450/100 000 and 500–740/100 000 in children aged <5 years [23, 27] and these figures are higher than the range estimated by the present study (366–420/100 000). These previous Canadian studies both involved retrospective review of hospital records for multiple AGE codes, including unspecified AGE-associated codes, and used modelling techniques to attribute a proportion to rotavirus. Both of these previous studies examined hospital records solely from a small area of the province of Quebec and thus may not be representative of national trends in hospitalization rates. Moreover, they did not consider or control for norovirus infections in their estimates. These previous studies did demonstrate substantial year-to-year variations in rotavirus hospitalizations, a pattern also observed in our estimates. This variation has also been observed in the United States and may be partially explained by genetic variation in circulating strains of virus [28].

We also observed considerable annual variability in the estimated number of hospitalizations for norovirus infection, which is consistent with other studies. Circulating strains of norovirus are known to evolve, with new strains emerging every few years and causing increased numbers of illnesses [29]. For example, in 2006/2007 two new strains emerged in Canada and the rest of the world causing a substantial increase in the number of observed outbreaks of norovirus infection [22, 30].

Analysis of hospitalization records indicated that 46% of norovirus patients developed symptoms after admission to hospital. This finding is in line with the literature as a high number of outbreaks are known to occur in hospital settings [9]. However, this is the first report, to our knowledge, to quantify the rate of nosocomial transmission in Canadian hospitals. Nosocomial norovirus infection likely extends the hospital stays of many of these patients who are initially hospitalized for other conditions. The estimated rate of nosocomial transmission was, however, much lower for rotavirus at 6% in all age groups and only 5% in patients aged <5 years. This is likely an underestimate as previous studies that used active surveillance to identify cases found substantially higher nosocomial rates of 14–51% in the <5 years age group [31, 32].

There are potential limitations of this study. The regression models considered only norovirus or rotavirus infections and not a wide range of other enteric viruses known to cause gastroenteritis. For instance, enteric viruses such as sapovirus, astrovirus and adenovirus are increasingly recognized as potentially important contributors to enteric illness [33], and sapovirus and astrovirus infections also tend to increase in the winter months [34, 35]. Therefore it is possible that proportions of the cause-unspecified discharges attributed to norovirus and rotavirus in our study are attributable to these other enteric viruses. Second, we assumed that a fixed multiplier could be applied to the number of coded hospitalizations to give a total number attributed to norovirus and rotavirus could be used to accurately estimate total hospitalizations for these viral infections. This approach may not take into account seasonal trends in testing practices or changes over time. Finally, only a few years of data were available for this study limiting the ability to assess any long-term trends in the number of infections.

Also of note, two rotavirus vaccines are approved and recommended for use in Canada; Rotarix™ was approved in 2008 and RotaTeq^®^ in 2006 [36]. However, only one province, with 0·4% of the Canadian population, had implemented a provincially funded vaccination initiative during the time-frame of this study (December 2010). It is unknown how many children were vaccinated during the study period, either through this provincially funded programme or through private purchases of vaccine. Since only a few years of data were analysed, and given the year-to-year variability observed, the effect of these vaccines could not be discerned. Analysis of hospitalization records beyond 2011 could provide insight into the effectiveness of the rotavirus vaccination programmes.

The total hospitalization costs were calculated based on the cost of a case with a most responsible diagnosis code of either norovirus or rotavirus, as a result the same cost was applied to community-acquired and nosocomial infections. Data limitations prevented the separation of costs for patients with a diagnosis of enteric viral infection post-admission. The underlying assumption of this approach was that the additional cost of an individual acquiring a virus in hospital is similar to the cost of an individual being hospitalized for an enteric viral infection. In addition, it should be noted that the cost estimates reported in this paper only reflect direct costs to hospitals and do not include costs associated with visits to physicians' offices, emergency rooms or other healthcare costs. Our estimates indicate that although rotavirus hospitalizations are more common, the total cost was higher for norovirus hospitalizations. This likely reflects the higher cost per hospitalization associated with the elderly which make up the majority of norovirus hospitalization cases in our database.

These estimates are the first comprehensive and age-specific assessment of norovirus and rotavirus hospitalizations in Canada. They provide data to understand the true scope of the burden associated with enteric viruses and can be used to assist public health decision-making.
